# Precision medicine in diagnosis, prognosis, and disease monitoring of bone and soft tissue sarcomas using liquid biopsy: a systematic review

**DOI:** 10.1007/s00402-024-05711-w

**Published:** 2025-01-11

**Authors:** Maria Anna Smolle, Markus G. Seidel, Karl Kashofer, Bernadette Liegl-Atzwanger, Patrick Sadoghi, Daniel A. Müller, Andreas Leithner

**Affiliations:** 1 Department of Orthopaedics and Trauma, Medical University of Graz, Auenbruggerplatz 5, 8036 Graz, Austria; 2 Research Unit for Cancer and Inborn Errors of the Blood and Immunity in Children, Division of Paediatric and Adolescent Haematology/Oncology, Department of Paediatric and Adolescent Medicine, Medical University of Graz, Auenbruggerplatz 38, 8036 Graz, Austria; 3 Diagnostic and Research Institute of Pathology, Medical University of Graz, Neue Stiftingtalstraße 6, 8010 Graz, Austria; 4 Balgrist University Hospital, University of Zurich, Forchstrasse 340, 8008 Zurich, Switzerland

**Keywords:** Liquid biopsy, Soft tissue sarcoma, Bone sarcoma, Circulating tumour DNA

## Abstract

**Introduction:**

Liquid biopsy as a non-invasive method to investigate cancer biology and monitor residual disease has gained significance in clinical practice over the years. Whilst its applicability in carcinomas is well established, the low incidence and heterogeneity of bone and soft tissue sarcomas explains the less well-established knowledge considering liquid biopsy in these highly malignant mesenchymal neoplasms.

**Materials and methods:**

A systematic literature review adhering to the PRISMA guidelines initially identified 920 studies, of whom 68 original articles could be finally included, all dealing with clinical applicability of liquid biopsy in sarcoma. Studies were discussed within two main chapters, i.e. translocation-associated and complex-karyotype sarcomas.

**Results:**

Overall, data on clinical applicability of liquid biopsy in 2636 patients with > 10 different entities of bone and soft tissue sarcomas could be summarised. The five most frequent tumour entities included osteosarcoma (n = 602), Ewing sarcoma (n = 384), gastrointestinal stromal tumour (GIST; n = 203), rhabdomyosarcoma (n = 193), and leiomyosarcoma (n = 145). Of 11 liquid biopsy analytes, largest evidence was present for ctDNA and cfDNA, investigated in 26 and 18 studies, respectively.

**Conclusions:**

This systematic literature review provides an extensive up-to-date overview about the current and potential future uses of different liquid biopsy modalities as diagnostic, prognostic, and disease monitoring markers in sarcoma.

**Supplementary Information:**

The online version contains supplementary material available at 10.1007/s00402-024-05711-w.

## Introduction

Precision medicine in oncology aims at improving diagnosis, treatment, and follow-up of cancer patients by identifying potential diagnostic, predictive and prognostic markers via genomic and molecular analyses [1, 2]. Usually they are discovered by pathologists upon histopathological examination of tumour tissue [3]. Liquid biopsy (LB) allows for detection of these very biomarkers in any biological fluid (usually blood). Due from its non-invasive nature, LB is ideal for application at many time points, enabling disease monitoring [1].

Different sources may be used as LB markers, including microRNA (miRNA), messenger RNA (mRNA), cell-free RNA (cfRNA), cell-free DNA (cfDNA), extracellular vesicles (EVs) as tumour-educated platelets (TEPs) and exosomes, circulating tumour DNA (ctDNA), circulating tumour cells (CTCs), and circulating cell-free mitochondrial DNA (ccf mtDNA) [1, 4].

Although knowledge on LB in cancer patients is largely based on carcinomas, information on their use in sarcomas has evolved recently and has promising aspects. However, due to the low incidence and heterogeneity of sarcomas, pertinent knowledge is based on the analysis of small or mixed cohorts. From a genetical point of view, sarcomas may be broadly differentiated into simple and complex karyotype subtypes [5], and some harbour a constant, tumour-defining driver fusion oncogene as main target for the application of LB [6, 7].

To shed light on the growing body of experience and various trends of LB in sarcoma, we performed a systematic literature review to summarise current knowledge on the applicability of different LB approaches as an example of precision medicine in clinical practice regarding diagnosis, treatment, and disease-monitoring of patients suffering from bone and soft tissue sarcomas (STS).

## Methods/literature search

A systematic literature review in PubMed was performed according to the *Preferred Reporting Items for Systematic Reviews and Meta-Analyses* (*PRISMA*) guidelines, including all original English and German articles (including case reports) published until 08.02.2023 and dealing with LB in sarcoma. The following search terms were used, without retrospective time restriction: LB AND sarcoma, LB AND soft tissue sarcoma, LB AND bone sarcoma, LB AND osteosarcoma, LB AND Ewing sarcoma, LB AND chondrosarcoma, cell-free DNA AND sarcoma AND LB, cell free DNA AND sarcoma AND biopsy (Supplemental Table 1). Preclinical and experimental studies, reviews, expert opinions, letters, and consensus statements were excluded. Level of evidence was defined according to the *Oxford Centre for Evidence-Based Medicine* (*OCEBM*) [8].

## Results

From initially 920 studies, 68 articles were finally included in this systematic review, all dealing with clinical application of LB in sarcoma (see PRISMA flowchart Supplemental Fig. 1 for detailed selection process).

**Fig. 1:**
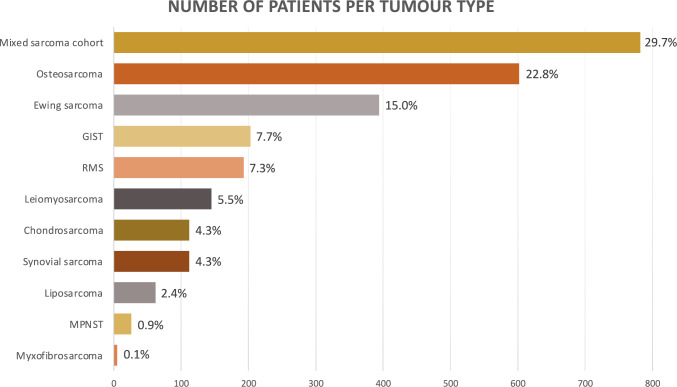
Number of patients per tumour type investigated

Studies were summarised in 2 chapters (translocation-associated sarcomas, complex-karyotype sarcomas), and 11 categories according to the prevailing histological subtype. Studies reporting on several sarcoma subtypes combined were summarised in a separate chapter. Altogether, data of 2 636 sarcoma patients were available (Fig. 1).

Apart from several bone and STS (*N* = 782; 29.7%), osteosarcoma (*N* = 602; 22.8%), Ewing sarcoma (ES; *N* = 384; 15.0%), gastrointestinal stromal tumour (GIST; *N* = 203; 7.7%), rhabdomyosarcoma (RMS; *N* = 193; 7.3%) and leiomyosarcoma (*N* = 145; 5.5%) were most common (Fig. 1).

### Translocation-associated sarcomas

#### Ewing sarcoma

The largest number of studies reported on the clinical application of LB in ES, an ideal candidate for LB analysis given the fact of pathognomic “driver” mutations [9]. Authors investigated different pertinent sources of LB, including ccf mtDNA, mRNA, miRNA, ctRNA, cfDNA, ctDNA, and CTCs. Overall, cfDNA- and ctDNA-based methods seem most promising in ES, albeit other LB sources look also promising (Table 1).

**Table 1:** Studies reporting on use of blood-based liquid biopsy in translocation-associated sarcomas

Study (alphabetical order)	Disease (patient number)	Material	Analysis	Relevance	OCEBM
*Ewing sarcoma*					
Abbou S 2022 Mod Pathol [30]	**ES family tumours (n = 8)**	CTCs	Fusion-specific tiered multiplexed PCR amplification followed by ddPCR	Fusion-specific multiplexed PCR amplification prior to ddPCR has higher sensitivity to detect *EWSR1* fusions in ES family tumours	IV
Allegretti M 2018 Ther Adv Med Oncol [16]	**ES (*N* = 4)**^a^	ctRNA	RT-qPCR, dPCR	*EWS::FLI1* detectable with custom primer sets in tissue (FFPE) and plasma; ctRNA levels correlate with metabolic tumour uptake as visible on PET imaging	III
Benini S 2018 Cancer Manag Res [29]	**ES (*N* = 18)** Healthy controls (*N* = 9)	CTCs	Immunomagnetic separation with microbeads and CD99 monoclonal antibodies; RT-qPCR	CTCs detectable in blood of ES patients	III
Bodlak A 2021 J Mol Diagn [21]	**ES (*N* = 5)**	ctRNA, ctDNA	ddPCR	ctDNA detection superior to ctRNA detection in ES	IV
Cahn F 2022 Cancers [24]	**ES (*N* = 5)** Osteosarcoma (*N* = 7) STS (*N* = 9) Other paediatric cancers (*N* = 24)	cfDNA	Foundation One^®^liquid CDx NGS panel (F1LCDx; 324 genes)	Specific mutations detectable in cfDNA from whole blood of ES (80%), osteosarcoma (57%) and STS (33%) patients	III
Colletti M 2018 Epigenomics [15]	**DSRCT (*N* = 3)** Healthy controls (*N* = 4)	miRNA	qPCR	14 miRNAs highly dysregulated in DSRCT patients in comparison to healthy controls, of these 8 highly upregulated (miR-34a-rp, miR-885-5p, miR-122-5p, miR-22-3p, miR-143a-3p, miR-497-5p, miR-324-5p, miR-365a-3p), 6 highly downregulated (miR-342-3p, miR-376c-3p, miR-150-5p, miR-543, miR-195-5p, miR-93-3p) in DSRCT	IV
Crow J 2022 Biomark Insights [13]	**ES family tumours (*N* = 5)** Osteosarcoma (*N* = 2) RMS (*N* = 2) Non-cancer controls (*N* = 4)	sEVs (miRNA)	62 exosome-miRNA panel	4 out of 5 ES family tumours detected via exosome-miRNA; patients with ES family tumours have nearly threefold increased miRNA levels in comparison to RMS, osteosarcoma, or non-cancer controls	III
Ferreira EN 2016 Hum Genomics [23]	**DSRCT (*N* = 1)**	ctDNA	Mate-paired WGS, ddPCR	Personalised biomarker based on *EWS::WT1* gene fusion developed to monitor ctDNA levels during follow-up (no ctDNA detected, in accordance with clinical/radiological remission)	V
George SL 2019 Eur J Cancer [17]	**ES (*N* = 2)**^b^ RMS (*N* = 2)^b^ Osteosarcoma (*N* = 1)^b^ Other paediatric malignancies (*N* = 7)^b^	ctDNA	ctDNA-specific NGS panel	Mutant ctDNA detectable in 2/2 ES patients, 2/2 RMS patients (in one below predefined limit of detection for clinical reporting), and 1 osteosarcoma patient	III
Klega K 2018 JCO Precis Oncol [22]	**ES (*N* = 11)** Osteosarcoma (*N* = 10) Alveolar RMS (*N* = 7) Other paediatric malignancy (*N* = 18)	ctDNA	ULP-WGS; hybrid capture sequencing assay (TranSS-Seq)	ctDNA detectable in 4/11 and 10/11 ES patients using ULP-WGS and TranSS-Seq, respectively	III
Krumbholz M 2016 Clin Cancer Res [6]	**ES (*N* = 20)**	ctDNA	ddPCR	*EWSR::FLI1* and *EWSR::ERG* fusion detectable in ctDNA from 18/20 patients using patient-specific primers; ctDNA levels correlate with tumour volume; most ctDNA levels decrease upon chemotherapy; increasing ctDNA levels indicate relapse	II
Krumbholz M 2021 Clin Cancer Res [19]	**ES (*N* = 102)**	ctDNA	ddPCR	Detectable fusion-positive ctDNA levels after second induction chemotherapy cycle indicative of poor EFS and OS	II
Lee SY 2018 Exp Ther Med [31]	**Uterine ES (*N* = 1)**	CTC, ctDNA	CTC enrichment kit (stained with epCAM/CK, Pan-CK, CD45, DAPI, vimentin-FITC, vimentin-PE antibodies), RT-qPCR	*FGFR3/*4 mutations detectable in CTCs; *FGFR4* and *HRAS* mutations found in cfDNA	V
Peneder P 2021 Nat Commun [27]	**ES (*N* = 95)** Other paediatric sarcoma (*N* = 31) Healthy controls (*N* = 22)	cfDNA	Deep WGS, ddPCR (targeting *EWS::ETS* fusion oncogene); ichorCNA (for CNA quantification), computational methods	ctDNA detectable without specific mutations or CNAs; epigenetic signatures allow for differentiation between tumour types; monitoring of CNAs and disease progression	III
Pfleiderer C 1995 Int J Cancer [10]	**ES family tumours (*N* = 16)**	CTCs	RT-PCR	CTCs detectable in 1/16 patients with RT-PCR (i.e. presence of *EWS::FLI1* RNA)	III
Samuel G 2020 Oncotarget [14]	**ES (*N* = 10)** Healthy controls (*N* = 6)	sEVs (mRNA)	Immunoprecipitation with membrane-bound proteins CD99/MIC2 and NGFR to enrich for tumour-derived sEVs	*EWS::ETS* fusion transcripts detectable in EVs of 7/10 ES patients using immunoprecipitation with two membrane-bound proteins (CD99/MIC2, NGFR); more reliable detection of tumour-derived sEVs possible with combined immune-isolation than with a single marker (CD9, member of tetraspanin superfamily)	IV
Schmidkonz C 2020 Eur J Nucl Med Mol Imaging [7]	**ES (*N* = 20)**	ctDNA	ddPCR	ctDNA levels correlate with PET-parameters (SUVmax, SUVmean, wb-MTV, wb-TLG); non-detectable ctDNA levels after the 2nd cycle of induction chemotherapy significantly associated with metabolic and biochemical response after 5th cycle; complete remission after 5th cycle of induction chemotherapy predictive of remission during follow-up	II
Seidel MG 2022 Front Pediatr [18]	**ES (*N* = 6)**	cfDNA	WGS, ddPCR	Detection of *EWS::FLI1* mutations in ctDNA of ES patients with patient-specific fusion breakpoint primers; correlation of ctDNA levels with clinical progression	III
Shah AT 2021 Mol Cancer Ther [20]	**ES (*N* = 8)** Osteosarcoma (*N* = 4) RMS (*N* = 4) Synovial sarcoma (*N* = 1)	ctDNA	Cancer Personalized Profiling by deep Sequencing (CAPP-Seq), ichorCNA	Translocations detected in pre-treatment plasma samples of 13/16 paediatric sarcoma patients (81.3%); pre-treatment CNAs in 7/17 patients; ctDNA levels correlate with metastatic status and clinical response; rising ctDNA levels detectable prior to clinically apparent relapse	IV
Shukla NN 2017 JCO Precis Oncol [25]	**ES (*N* = 11)** DSRCT (*N* = 6)	cfDNA	ddPCR, disease-tailored targeted hybridization capture-based NGS panel	*EWSR1* fusions identified in 100% of ES and 83% of DSRCT cfDNA samples by ddPCR; *EWSR1* fusions detected in 91% and 67% of ES and DSRCT cfDNA samples by NGS; additional *TP53* and *STAG2* mutations identified in cfDNA by NGS	III
Van Paemel R 2022 Eur J Caner [26]	**ES (*N* = 9)** Osteosarcoma (*N* = 10) RMS (*N* = 10) Other paediatric malignancy (*N* = 99)	cfDNA	sWGS	Good concordance between CNAs in tissue DNA and cfDNA of paediatric cancer patients; higher cfDNA/HMW ratio corresponds with better agreement between tissue and cfDNA CNAs; presence of CNAs in plasma but not tissue samples (or vice versa) indicates spatial heterogeneity	III
Yu M 2012 Arch Med Res [11]	**ES (*N* = 25)** Healthy controls (*N* = 20)	ccf mtDNA	rtPCR	ccf mtDNA levels are lower in ES than healthy controls; measurement of ccf mtDNA levels allow for discrimination between healthy controls and ES patients	III
*Rhabdomyosarcoma*					
Eguchi-Ishimae M 2019 Genes Chromosomes Cancer [34]	**Alveolar RMS (*N* = 1)**	cfDNA	qPCR	cfDNA harbouring *PAX3::FOXO1* fusion serially detected in blood stream of patient	V
Lak NSM 2023 JCO Precis Oncol [35]	**RMS (*N* = 57)** Alveolar (*N* = 22) Embryonal (*N* = 40) Other (*N* = 3)	ctDNA	shWGS, cfRRBS, ddPCR	ctDNA detected in 16/30 patients with shWGS, and 24/26 with cfRRBS; presence of *RASSF1A-M* (detected with ddPCR) associated with poor EFS	II
Ruhen O 2022 JCO Precis Oncol [36]	**RMS (*N* = 28)** Alveolar (*N* = 13) Embryonal (*N* = 9) Other (*N* = 1)	ctDNA	ddPCR, panel sequencing, WES	ctDNA found in 14/18 pre-treatment samples with ddPCR, and 7/7 samples with sequencing; higher ctDNA levels in patients with metastasis, unfavourable tumour site, lymph node status; ctDNA levels reflect course of disease over time (correlation with tumour burden and disease status)	III
Stegmaier S 2022 Pediatr Blood Cancer [37]	**Alveolar RMS (*N* = 65)** Synovial sarcoma (*N* = 15)	cfRNA	RT-qPCR	Initial liquid biopsy negative for *PAX::FOXO1* fusion in alveolar RMS patients with localised disease, but positive in 62% (*N* = 18) of metastatic cases; 5/48 samples collected during therapy and follow-up positive for *PAX::FOXO1* fusion; none of synovial sarcoma samples positive for cfRNA	III
Tombolan L 2022 Mol Oncol [33]	**RMS (*N* = 17)** Alveolar (*N* = 6) Embryonal (*N* = 10) Botroyd (*N* = 1)	Peripheral blood: CTCs, cfDNA Bone marrow: DTCs	CTCs, DTCs: WGA cfDNA: ddPCR	At least 1 CTC found in 9/13 (69%) patients with localised disease, and in 4/4 (100%) patients with metastatic disease; at least one DTC identified in 10/13 (76.9%) and 2/2 (100%) patients with localised and metastatic disease, respectively; positive correlation between CTC and cfDNA amount; similar genetic alterations found in CTCs and cfDNA	III
*Synovial sarcoma*					
Eisenhardt AE 2022 Cancers [42]	**Synovial sarcoma (*N* = 25)**	ctDNA	NGS-based patient-specific breakpoint panel for *SS18::SSX1/2* fusion; patient-specific exome panel	Breakpoint ctDNA detected in 6/14 of blood samples; patient-specific exome panel in one patient revealed correlation of ctDNA levels with disease burden over time	III
Mihály D 2018 Diagn Pathol [41]	**Synovial sarcoma (*N* = 15)**	cfRNA	Nested PCR, ddPCR	1/15 liquid biopsy samples positive for *SS18::SSX1* fusion using ddPCR, 0/15 liquid biopsy samples positive for *SS18::SSX* fusion with nested PCR	III
Ogino S 2018 World J Gastroenterol [43]	**Gastric synovial sarcoma (*N* = 1)**	cfDNA	PCR	*SYT::SSX* fusion detected in preoperative, but not postoperative cfDNA	V
Przybyl J 2019 Diagn Pathol [39]	**Synovial sarcoma (*N* = 38)**	CTCs	Nested RT-PCR	*SS18::SSX1* fusion transcripts detected in 2/38 patients (based on CTCs)	III
Stegmaier S 2022 Pediatr Blood Cancer [37]	**Synovial sarcoma (*N* = 15)** Alveolar RMS (*N* = 65)	cfRNA	RT-qPCR	0% (0/15) of synovial sarcoma blood samples positive for *SYT::SSX1/2* fusion	III
Yokoo S 2021 Cancers [40]	**Synovial sarcoma (*N* = 17)** Healthy controls (*N* = 4)	EVs	Sandwich ELISA	Higher levels of MCT1 + CD9 + EVs in preoperative in comparison to postoperative samples	III
*Gastrointestinal stromal tumour*					
Bauer S 2021 Clin Cancer Res [46]	**GIST (*N* = 122)**^a^	ctDNA	NGS	*KIT/PDGFRa* mutation detectable in 93/122 liquid biopsy samples; of these, 25, 28 and 37 patients had 1, 2 of ≥ 3 *KIT* mutations, respectively; 3 patients had *PDGFRa* mutations	II
Jilg S 2019 Int J Cancer [45]	**GIST (*N* = 25)**	ctDNA	L-PCR, ddPCR	Tumour-specific *KIT* or *PDGFRa* mutant ctDNA found in 16/25 (64%) and 20/25 (80%) of patients with L-PCR and ddPCR; absolute number of ctDNA fragments and MAF (as assessed with ddPCR) correlated with tumour size and response status; less pronounced correlation between MAF and tumour size as well as response status	II
Johansson G 2021 Mol Cancer Ther [48]	**GIST (*N* = 32)**	ctDNA	qPCR, SiMSen-Seq panels	*KIT/PDGFRa* mutations detected at least once in 9/32 patients (based on 161 blood samples obtained perioperatively); correlation between presence of mutant ctDNA and large tumour size, increased cell proliferation rate; no patient with *KIT* mutant ctDNA preoperatively had measurable ctDNA postoperatively	III
Serrano C 2020 BMC Cancer [47]	**GIST (*N* = 18)**	ctDNA, cfDNA	NGS, ddPCR	Tumour-specific cfDNA mutations (*KIT/PDGFRa*) found in 6/21 blood samples with NGS, and in 9/21 blood samples with ddPCR; ctDNA shedding low; ctDNA detection more likely in patients with advanced disease	III

The disease group of interest is highlighted in bold

*ccf mtDNA* circulating cell-free mitochondrial DNA, *cfDNA* cell-free DNA, *cfRNA* cell-free RNA, *cfRRBS* cell-free reduced representation bisulphite sequencing, *CNA* copy-number alteration, *CTCs* circulating tumour cell, *ctDNA* circulating tumour DNA, *ctRNA* circulating tumour RNA, *ddPCR* digital droplet polymerase chain reaction, *dPCR* chip-based digital PCR, *DSCRT* desmoplastic small round cell tumour, *DTCs* disseminated tumour cells, *EFS* event-free survival, *ELISA* enzyme-linked immunosorbent assay, *ES* Ewing sarcoma, *EV* extracellular vesicle, *FFPE* formalin-fixed paraffin-embedded, *FGFR3/4* fibroblast growth factor receptor 3/4, *FOXO1* forkhead box O1, *GIST* gastrointestinal stromal tumour, *HMW* high molecular weight, *KIT* v-kit Hardy-Zuckerman 4 feline sarcoma viral oncogene homolog, *L-PCR* allele-specific ligation polymerase chain reaction, *LC/MS* liquid chromatography mass spectrometry, *MAF* mutant allele frequency, *MTC1* monocaboxylate transporter 1, *NGS* next generation sequencing, *OCEBM* Oxford Centre of Evidence-based Medicine, *OS* overall survival, *PAX3* paired box 3, *(q)PCR* (quantitative) polymerase chain reaction, *PDGFRa* platelet derived growth-factor alpha, *PET* positron-emission tomography, *RASSF1A-M* methylated Ras association domain family member 1A, *RMS* rhabdomyosarcoma, *RT-qPCR* real-time quantitative polymerase chain reaction, *sEVs* small extracellular vesicles, *shWGS* shallow whole genome sequencing, *SiMSen-Seq* simple multiplexed PCR-based barcoding of DNA for sensitive mutation detection using sequencing, *SSX* 1/2 SSX family member 1/2, *STAG2* Stromal Antigen 2, *STS* soft tissue sarcoma, *SUV* standardized uptake value, *sWGS* shallow whole genome sequencing, *SYT* synaptotagmin (SS18), *TEM* transmission electron microscopy, *TP53* Tumour Protein 53, *TranSS-Seq* Translocation-Specific Sarcoma Sequencing assay, *ULP-WGS* ultralow passage whole-genome sequencing, *wb-MTV* whole-body metabolic tumour volume, *wb-TLG* whole-body total lesion glycolysis, *WES* whole exome sequencing, *WGA* whole genome amplification, *WGS* whole-genome sequencing

^a^Patients with liquid biopsy samples available

^b^Patients with ctDNA analysis available

As early as in 1995, Pfleiderer et al*.* [10] tried to detect *EWS::FLI1* fusions by applying RT-PCR on RNA extracted from peripheral blood nucleated cells of 16 ES family tumour patients. They were successful in 1 individual [10].

According to Yu et al*.* [11], levels of ccf mtDNA are significantly lower in ES plasma samples than in healthy controls [12]. Serum ccf mtDNA to distinguish between ES patients and healthy individuals reached a sensitivity and specificity of 76.1% and 64.8%, respectively [11].

Via a 62 exosome-microRNA (miRNA) panel, Crow et al*.* correctly classified 4/5 ESs using plasma-derived small extracellular vesicles (sEVs) [13]. Notably, miRNA levels were nearly threefold higher in ES plasma samples as compared to those from osteosarcoma, RMS, and non-cancer patients [13].

Samuel et al*.* [14] likewise used sEVs as a LB analyte in ES. Immunoprecipitation with two membrane-bound proteins (CD99/MIC2, NGFR) allowed for reliable identification of tumour-derived sEVs [14]. In 7/10 ES samples analysed, *EWS::ETS* fusion transcripts were found with qRT-PCR in isolated sEVs [14].

Colletti et al*.* [15], compared miRNA expression profiles of 3 desmoplastic small round cell tumour (DSRCT)-patients with those of 4 healthy controls using qPCR. Of 14 miRNAs identified as significantly dysregulated (6 down- and 8 upregulated) in DSRCT as compared with controls (Table 1) [15].

Allegretti et al*.* [16] analysed tissue and plasma samples of six and four ES patients, respectively, with RNA extraction and RT-qPCR, as well as dPCR towards presence of circulating tumour RNAs (ctRNAs). They detected two prevalent types of *EWS::FLI1* rearrangements with two custom primer sets likewise in tissue and ctRNAs, regardless of patient-specific DNA break-points in *EWS::FLI1* [16]. ctRNA levels positively correlated with metabolic tumour uptake on positron emission tomography (PET) scans [16].

Similarly, Schmidkonz et al. [7] monitored ctDNA in plasma of 20 ES patients, and correlated their levels with PET-parameters as standardized uptake value (SUV) max, SUVmean, whole-body metabolic tumour volume (wb-MTV), and whole-body total lesion glycolysis (wb-TLG) [7]. In 16/17 patients with non-detectable ctDNA after the 2nd chemotherapeutic induction cycle, a complete biochemical and metabolic response was evident following the 5th induction cycle [7] which had a positive predictive value of 88% for long-term disease remission [7].

George et al*.* [17] analysed mutant ctDNA in peripheral blood of 12 paediatric patients with extracranial tumours (including 2 ES, 2 RMS, 1 osteosarcoma) using a ctDNA-specifc next-generation sequencing (NGS) panel covering mutations identified in FFPE samples [17]. They found mutant ctDNA in both ES patients [17].

Seidel et al*.* identified patient-specific *EWS::FLI1* fusion breakpoints via whole genome sequencing (WGS) in 6 ES cases [18]. Plasma samples before, during and after treatment were tracked for these genes with digital droplet PCR (ddPCR), revealing that changes in ctDNA levels within single patients correlated with course of disease, whilst a large variability was observed between different patients [18].

Similarly, Krumbholz et al*.* [6] applied ddPCR on plasma of 20 ESs using patient-specific fusion gene primers for *EWSR1::FLI1* or *EWSR1::ERG* rearrangements. In 18/20 patients, ctDNA copies were detected. The copy number correlated with tumour volume and relapse [6]. The follow-up article by the same group included results from 102 ES patients (489 plasma samples) analysed for mutant ctDNA with ddPCR [19]. Persistence of fusion-positive ctDNA after two induction chemotherapy cycles was a strong predictor for poor event-free and overall survival [19].

Shah et al*.* [20], applied a Cancer Personalized Profiling by deep Sequencing (CAPP-Seq) to detect translocations typically found in paediatric sarcomas [20]. Of 17 paediatric sarcoma patients (including 8 with ES), translocations were identified in ctDNA of 13/16 plasma samples [20]. ctDNA levels correlated with metastatic status and clinical response, with reascending ctDNA levels detectable prior to clinically apparent relapse [20].

Bodlak et al*.* [21] found that ctDNA could more reliably be detected than ctRNA in the plasma in 5 ES patients. Although fusion transcript ctRNA assays enabled detection of *EWS::ERG* and *EWS::FLI1* transcripts, copy number burden was significantly lower than in breakpoint-specific ctDNA assays [21]. As the overall cfRNA burden did not correlate with ctRNA levels, the authors suggested that most cfRNA is non-tumour-derived, partially explaining the poor performance of the ctRNA assay [21].

Klega et al*.* [22] compared two methods (ultralow passage whole genome sequencing [ULP-WGS]; unique hybrid capture sequencing assay, termed “Translocation-Specific Sarcoma Sequencing assay” [TranSS-Seq]) to measure blood-based ctDNA levels in paediatric tumours, including ESs. ctDNA was identified in 4/11 ES patients using ULP-WGS, and in 10/11 samples when applying TranSS-Seq [22].

Ferreiera et al. [23] used mate-paired WGS to define a specific *EWS::WT1* translocation, and ddPCR to detect it in plasma of a DSRCT patient. Notably, no *EWS::WT1* mutant ctDNA was found during follow-up, in accordance with no signs of active disease on radiology [23].

Using the Foundation One^®^ Liquid CDx NGS panel, Cahn et al*.* identified *EWSR1* fusions in cfDNA of blood from 4/5 ES patients [24].

Shukla et al. [25] used patient-specific fusion gene primers and ddPCR to identify *EWSR1* fusions in cfDNA of plasma samples from 11 and 6 patients with ES and DSRCTs, respectively. Sensitivity to detect *EWSR1* fusions in plasma samples was higher with ddPCR than with NGS (100% vs. 91% for ES; 83% vs. 67% for DSRCT) [25].

Using shallow WGS, van Paemel et al. detected corresponding copy-number alterations (CNAs) in tissue and cfDNA plasma samples of paediatric cancer, including ES, osteosarcoma, and RMS [26]. Reasons for CNA discordance between tissue and plasma were high molecular weight DNA (HMW; > 700 base pairs [bp]) or low cfDNA ratio (< 700 bp) [26].

Peneder et al*.*, analysed 241 deep WGS profiles deriving from blood samples of 95 ES, 31 patients with other paediatric sarcomas, and 22 healthy controls [27]. Following genetic analysis with three approaches, they studied global DNA fragmentation patterns, as they reflect chromatin profiles of cfDNA-releasing cells [28]. ES patients had shorter cfDNA fragments than healthy controls [27]. Filtering for short fragment cfDNA increased sensitivity to detect ES-specific CNAs [27].

Benini et al*.* [29], isolated ES -derived CTCs from blood via immunomagnetic separation using microbeads and CD99 monoclonal antibodies. Presence of CTCs was confirmed by RT-qPCR targeting *EWSR1::FLI1* type 1/2 or *EWSR1::ETS*-related gene transcripts type 1 and 9e [29].

Alternatively, Abbou et al*.* [30], isolated CTCs from peripheral blood by enrichment with Celsee PREP 100 and CTC enrichment Kit or ScreenCell MB kit. Thereafter, RNA was extracted from enriched CTCs, and pre-amplified with a fusion-specific multiplex PCR prior to ddPCR (tiered multiplexed amplification). This combined approach improved the sensitivity for pathognomic fusions [30].

Lee et al*.* [31], isolated epithelial cell adhesion molecule (EpCAM)-positive CTCs from plasma samples in a 16-year-old female patient with uterine ES, in whom *fibroblast growth factor 3/4 (FGFR3/4*) mutations, as well as an *EWSR1* gene sequence were detected [31]. In addition, ctDNA analysis of the same patient revealed *HRAS* and *FGFR4* mutations [31].

#### Rhabdomyosarcoma

Up to 90% of alveolar RMS patients harbour a *Paired Box 3/7 (PAX3/7)::Forkhead Box O1 (FOXO1)* fusion [32] making alveolar RMS an ideal candidate for LB. Different sources for LB in alveolar and embryonal RMS have been investigated, including CTCs, cfDNA, ctDNA, and cfRNA. Table 1.

Tombolan et al*.* [33], analysed peripheral blood and bone marrow samples of 17 RMS patients towards CTCs, cfDNA, and disseminated tumour cells (DTCs). For CTC and cfDNA analysis, whole genome amplification (WGA) and ddPCR with specific primers were used [33]. They found at least one CTC in 9/13 and 4/4 patients with localised and metastatic disease, respectively [33]. CTC and cfDNA levels correlated positively, with similar genetic alterations in both analytes [33].

Eguchi-Ishimae et al*.* [34] investigated cfDNA in a 15-year-old RMS patient. With qPCR, cfDNA harbouring the pathognomic *PAX3::FOXO1* fusion was identified, and cfDNA levels mirrored disease burden during follow-up [34].

Lak et al*.* [35] performed shallow WGS (shWGS) and cell-free reduced representation bisulphite sequencing (cfRRBS) to identify cfDNA in peripheral blood of alveolar (*N* = 22), embryonal (*N* = 40), and other (*N* = 3) RMS patients. Moreover, they aimed at detecting *methylated Ras association domain family member 1A* (*RASSF1A-M*) with ddPCR [35]. shWGS and cfRRBS detected ctDNA in 16/30 and 24/26 patients, respectively [35]. Presence of *RASSF1A-M* (in 21/57 patients) was associated with a poorer event-free survival [35].

Ruhen et al*.* [36] used ddPCR, whole exome sequencing (WES), and panel sequencing to detect cfDNA and ctDNA in blood samples of 28 RMS patients. Pre-treatment samples showed a tumour-specific variant in 14/18 patients, including *PAX3::FOXO1* fusion in 10/18 [36]. Furthermore, WES in 7 patients with matched germline, tumour and baseline cfDNA was performed, enabling ctDNA identification in all 7 blood samples. ctDNA levels were higher in patients with poor prognosis and correlated with tumour burden during follow-up [36].

cfRNA stabilised in exosomes constitutes another potential LB analyte without requiring cumbersome methods to prevent molecule degradation [37, 38]. Stegmaier et al*.* [37] isolated cfRNA from blood of 65 alveolar RMS patients positive for tumour-specific fusions. In primary localized RMS RT-qPCR showed no tumour-specific fusions (*PAX3/7::FOXO1*) in cfRNA at baseline, compared to 62% in primary metastatic patients [37].

#### Synovial sarcoma

The prevalence of tumour-specific fusion transcripts in peripheral blood of synovial sarcoma pateints seems low, the material analysed notwithstanding [37, 39, 40] Table 1.

Stegmaier et al*.* [37] searched for tumour-specific fusions in cfRNA in 15 synovial sarcoma patients. None of the blood samples with known *synaptotagmin* (*SYT; SS18*)::*SSX family member 1/2* (*SSX1/2*) fusions in the primary tumour had detectable cfRNA [37].

Similarly Mihály et al. [41], identified *SS18::SSX1* fusion in only 1/15 synovial sarcoma patients with ddPCR. Using nested PCR, this fusion could not be detected in any of the 15 samples [41].

Eisenhardt et al*.* [42] used an NGS-based patient-specific breakpoint panel for *SS18::SSX1/2* fusions to detect ctDNA in blood of 15 synovial sarcoma patients, retrieving positive results in 6/15 individuals [42].

Ogino et al*.* [43] detected *SYT (SS18)::SSX* fusion transcripts in cfDNA of a patient with gastric synovial sarcoma in preoperative, but not in postoperative blood samples.

Based on the results of a mouse model, Yokoo et al. [40], used ELISA to identify monocarboxylate transporter 1 (MCT1) + CD9 + EVs as surface marker in 17 synovial sarcoma patients. MCT1 + CD9 + EV levels were higher in preoperative than in postoperative blood samples, mirroring reduced tumour burden [40].

Like cfRNA and EVs, the analysis of CTCs towards tumour-specific fusions appears limited in synovial sarcoma. Przybyl et al*.* [39] found positive results in only 2/38 blood samples analysed with nested RT-PCR for *SS18::SSX1/2* fusions in CTCs.

#### Gastrointestinal stromal tumour

Between 85 and 90% of gastrointestinal stromal tumours (GIST) harbour a mutation of *v-kit Hardy-Zuckerman 4 feline sarcoma viral oncogene homolog* (*KIT*) or platelet-derived growth factor receptor alpha (*PDGFRa*) [44], as potential LB markers Table 1.

Jilg et al*.* [45] used allele-specific ligation PCR (L-PCR) and ddPCR to analyse *cKIT-* and *PDGFRa*-mutant ctDNA in blood samples of 25 GIST patients. With L-PCR, tumour-specific *cKIT* or *PDGFRa* mutations were found in cfDNA of 16/25 patients. In comparison, ddPCR detected *cKIT/PDGFRa* mutations in 20/25 individuals [45]. Absolute ctDNA fragment number and mutant allele frequency (MAF) as measured with ddPCR correlated with tumour size and treatment response [45].

Similarly, Bauer et al*.* [46] analysed *KIT* and *PDGFRa* mutations in ctDNA of 122 patients with GIST using the NGS-based LB assay Guardant360 (G*uardant Health Inc., Redwood City, CA, USA*) [46]. Mutant *KIT/PDGFRa* ctDNA was found in 93/121 patients (one failed analysis) [46]. Of these, 25, 28 and 37 patients had one, 2 and ≥ 3 *KIT* mutations, respectively, whereas 3 patients presented with *PDGFRa* mutations [46].

Serrano et al. [47] detected *KIT* and *PDGFRa* mutant ctDNA at low levels in 18 GIST patients (21 samples) using NGS and ddPCR. With NGS and ddPCR, they found known *KIT/PDGFRa* mutations in cfDNA of 6/21 and 9/21 samples, respectively [47]. Whilst no patient with localized GIST had detectable mutant ctDNA, detection rate increased in metastatic patients and in progressive disease [47].

Johansson et al*.* [48] developed panels targeting patient-specific *KIT* and *PDGFRa* mutations in GIST patients, termed “simple multiplexed PCR-based barcoding of DNA for sensitive mutation detection using sequencing” (*SiMSen-Seq*). SiMSen-Seq detected mutant ctDNA at least once in 9/32 patients (161 perioperative blood samples) [48]. All preoperatively positive patients had negative samples postoperatively, reflecting reduced tumour burden [48].

### Complex karyotype sarcomas

#### Osteosarcoma

The applicability of LB has been investigated in osteosarcoma, albeit to a lesser extent than in ES. Osteosarcoma lacks pathognomic “driver” mutations and presents with a complex karyotype [49]. Nevertheless, some promising results of LB have been published (Table 2).

**Table 2:** Studies reporting on use of blood-based liquid biopsy in complex-karyotype sarcomas

Study (alphabetical order)	Disease (patient number)	Material	Analysis	Relevance	OCEBM
*Osteosarcoma*					
Bao Q 2018 Ann Surg Oncol [55]	**Osteosarcoma (*N* = 4)** 2 patients with two matched samples (at diagnosis and metastasis) 2 matched patients (one with localised and one with metastatic disease) Osteosarcoma cell lines (*N* = 3)^b^	EVs	RNA sequencing	Increase of tumour mutation burden in metastatic EV samples as compared with non-metastatic (matched) samples	III
Cahn F 2022 Cancers [24]	**Osteosarcoma (*N* = 7)** ES (*N* = 5) STS (*N* = 9) Other paediatric cancers (*N* = 24)	cfDNA	Foundation One®liquid CDx NGS panel (F1LCDx; 324 genes)	Specific mutations detectable in cfDNA from whole blood of ES (80%), osteosarcoma (57%; mutations including *ART1*, *BRD4*, *NFKBIA*, *RAD21*, *ATRX*) and STS (33%) patients	III
Fujiwara T 2017 Oncotarget [51]	**Osteosarcoma (*N* = 10)** Age-matched non-osteosarcoma (*N* = 10) Healthy controls (*N* = 10)	miRNA	RT-qPCR	miR-25-3p levels significantly higher in osteosarcoma patients than controls; low miR-25-3p levels at diagnosis significantly associated with prolonged DM-free survival	IV
George SL 2019 Eur J Cancer [17]	**Osteosarcoma (*N* = 1)**^a^ ES (*N* = 2)^a^ RMS (*N* = 2)^a^ Other paediatric malignancies (*N* = 7)^a^	ctDNA	ctDNA-specific NGS panel	Mutant ctDNA (*TP53*) detectable in blood sample obtained at 2nd relapse in osteosarcoma patient, corresponding to *TP53* mutation identified in primary tumour tissue (with 1.4 years in-between)	III
Han Z 2022 Biosens Bioelectron [56]	**Osteosarcoma (*N* = 20)** Healthy controls (*N* = 20)	Exosomes	Size-dependent microfluidic filtration + SERS	Significantly higher levels of EpCAM, CD63, vimentin on plasma exosomes of osteosarcoma patients in comparison to controls; classification model based on these three markers identified osteosarcoma with high sensitivity (100%), specificity (90%) and accuracy (95%)	IV
Han Z 2021 iScience [57]	**Osteosarcoma (*N* = 40)** without metastasis (*N* = 20) with metastasis (*N* = 20) Healthy controls (*N* = 12)	Exosomes	MALDI-TOF MS	Plasma exosome analysis by MALDI-TOF MS enables differentiation between healthy controls and osteosarcoma patients, as well as osteosarcoma patients with and without metastases	IV
Han Z 2021 Analyst [58]	**Osteosarcoma (*N* = 15)** Healthy controls (*N* = 15)	Exosomes	SERS, MALDI-TOF MS	Osteosarcoma samples can be differentiated from healthy controls via exosomes profiling by MALDI-TOF MS and SERS, with combination of both methods achieving even higher accuracy	IV
Wang J 2023 Clin Cancer Res [59]	**Osteosarcoma (*N* = 67)**	Exosomal PD-L1	Immunogold labelling, ELISA	High exosomal PD-L1 levels significantly associated with poor disease-free and OS	II
Wang SN 2017 Mol Ther [52]	**Osteosarcoma (*N* = 102)** Healthy controls (*N* = 20)	miRNA	RT-qPCR	Osteosarcoma patients present with lower circulating miR-491 levels than healthy controls; low miR-491 levels independently associated with poor OS (gender, age, anatomical site, histological grade, Enneking grade, and response to chemotherapy included in multivariate model)	III
Xie XY 2021 Medicine (Balltimore) [50]	**Osteosarcoma (*N* = 243)** Healthy controls (*N* = 96)	miRNA	RT-qPCR	Circulating miR-26a-5p levels significantly higher in osteosarcoma patients than healthy controls; high miR-26a-5p levels associated with advanced disease and metastasis, as well as poor survival	III
Yang L 2020 Cancer Biother Radiopharm [53]	**Osteosarcoma (*N* = 42)** Healthy controls (*N* = 42)	lncRNA	RT-qPCR	Plasma MT1JP-levels downregulated in osteosarcoma patients in comparison to healthy controls	III
Zhang H 2017 Int J Oncol [54]	**Osteosarcoma (*N* = 23)** Localised (*N* = 10) Recurrent/metastatic (*N* = 13)	CTCs	FISH	Higher number of CTCs found in blood of recurrent/metastatic osteosarcoma patients in comparison to those with localised disease; ≥ 2 CTCs per 7.5 ml peripheral blood associated with worse PFS	III
*Chondrosarcoma*					
Gutteridge A 2017 Cancer Med [60]	**Chondrosarcoma (*N* = 29)**	ctDNA	ddPCR	ctDNA detected in 14/29 patient samples obtained preoperatively (identified with ddPCR assay for *IDH1* mutations); ctDNA levels correlated with tumour grade, i.e. never found in chondrosarcoma G1 (0/6), but present in 8/17, 2/2 and 4/4 patients with G2, G3 and dedifferentiated chondrosarcoma, respectively; reduction in ctDNA levels from pre- to postoperative observed in 7/9 patients	III
Lyskjaer I 2021 Mol Oncol [61]	**Chondrosarcoma (*N* = 83)**	ctDNA	ddPCR	ctDNA detectable in 31/83 patients preoperatively and 12/31 patients postoperatively (identified with ddPCR assay for *IDH1/2* or *GNAS* mutations); presence of ctDNA correlates with tumour grade (no mutations in atypical cartilaginous tumours or chondrosarcoma G1, mutations present in 3/4 and 10/13 patients with G3 and dedifferentiated chondrosarcoma; detectable pre- and postoperative ctDNA independently associated with worse overall survival	III
*Liposarcoma*					
Braig D 2019 Int J Cancer [67]	**Myxoid liposarcoma (*N* = 4)** WDLPS/DDLPS (*N* = 5) Other active STS (*N* = 55) STS in remission (*N* = 19) Healthy controls (*N* = 41)	cfDNA, ctDNA	RT-qPCR, ddPCR	cfDNA levels significantly higher in patients with active STS patients in comparison those in remission; *MDM2* amplification in cfDNA not sensitive enough to detect tumours in WDLPS/DDLPS; correlation of breakpoint t(12;16) and TERT C228T ctDNA levels with clinical course and tumour burden in myxoid liposarcoma (*N* = 4)	III
Fricke A 2018 Cancer Biomark [65]	**DDLPS (*N* = 6)** Lipoma (*N* = 5) Healthy controls (*N* = 4)	miRNA	RT-qPCR	miR-3613-3p and miR-4668-5p significantly upregulated in DDLPS in comparison to controls; in validation analysis, miR-3613-3p prevailed as significantly upregulated in DDLPS as compared with healthy controls or lipoma patients	III
Jung J 2016 Nat Commun [68]	**DDLPS (*N* = 20)**	cfDNA	NGS	*TP53* mutations detectable in cfDNA of DDLPS patients treated with MDM2-inhibitor; increasing *TP53* levels over time correlate with increase in tumour size	II
Kohama I 2021 Oncol Lett	**DDLPS (*N* = 17)** Osteosarcoma (*N* = 22) ES (*N* = 3)	miRNA	RT-qPCR	miR-1246, −4532 and −619-5p identified as potential markers in serum of patients with DDLPS	III
Przybyl J 2022 PLoS One [66]	**DDLPS (*N* = 3)** **WDLPS (*N* = 1)** Other soft tissue tumour (*N* = 11)	cfDNA	shWGS	*MDM2* amplification in cfDNA of 2/3 DDLPS patients; no *MDM2* amplification found in cfDNA of WDLPS patient, or patients with other soft tissue tumours	IV
*Leiomyosarcoma*					
Hemming ML 2019 JCO Precis Oncol [70]	**Leiomyosarcoma (*N* = 30)** Uterine (*N* = 16) Retroperitoneal (*N* = 8) Others (*N* = 6)	ctDNA	ULP-WGS, ichorCNA	ctDNA detected in 11/16 patients with disease progression and tumour burden > 5 cm; no ctDNA found in patients with stable disease; correlation between high ctDNA levels and large tumour size; CNAs found in primary tumour tissue correlate with those detected in plasma-based ctDNA	III
Madanat-Harjuoja LM 2022 Clin Cancer Res [71]	**Leiomyosarcoma (*N* = 98)**	ctDNA	ULP-WGS	ctDNA detected in 48/98 of pre-treatment samples, and 17/69 samples obtained after to chemotherapy cycles; poorer objective response and worse survival in patients with detectable ctDNA prior to treatment as well as after two chemotherapy cycles	III
Yokoi A 2019 Cancer Sci [72]	**Uterine leiomyosarcoma (*N* = 6)** **Uterine adenosarcoma (*N* = 2)** **Endometrial stromal sarcoma (*N* = 2)** **STUMP (*N* = 1)** Uterine leiomyoma (*N* = 18)	miRNA	miRNA microarray	miR-1246 and miR-191-5p expression profiles allow for differentiation between uterine sarcoma and uterine leiomyoma	III
*MPNST*					
Mattox AK 2022 Elife [74]	**MPNST (*N* = 12)** Plexiform neurofibroma (*N* = 9) Neurofibroma (*N* = 7) Healthy controls (*N* = 883)	ctDNA	ddPCR	Sensitivity to detect MPNST with genome wide aneuploidy scoring 33%; CNA-analysis improved sensitivity to 50%	III
Szymanski JJ 2021 PLoS Med [75]	**MPNST (*N* = 14)** Plexiform neurofibroma (*N* = 23) Healthy controls (*N* = 16)	cfDNA	ULP-WGS	Shorter cfDNA fragments and enrichment for tumour-derived cfDNA seen in MPNST in comparison to plexiform neurofibromas and healthy controls	III
*Myxofibrosarcoma*					
Morita T 2020 Sci Rep [76]	**Myxofibrosarcoma (*N* = 5)** Healthy controls (*N* = 9)	miRNA	Global miRNA profiling	miR-1260b levels significantly upregulated in myxofibrosarcoma patients in comparison to healthy controls; miR-1260b expression correlated with radiological tail-like pattern	IV

The disease group of interest is highlighted in bold

*ART1* ADP-Ribosyltransferase 1, *ARTX* Alpha Thalassemia/Mental Retardation Syndrome X-Linked, *BRD4* Bromodomain Containing 4, *cfDNA* cell-free DNA, *CNAs* copy number alteration, *CTCs* circulating tumour cells, *ctDNA* circulating tumour DNA, *DDLPS* dedifferentiated liposarcoma, *ddPCR* digital droplet polymerase chain reaction, *DM* distant metastasis, *ELISA* enzyme-linked immunosorbent assay, *EpCAM* epithelial cell adhesion molecule, *ES* Ewing sarcoma, *EVs* extracellular vesicles, *FISH* fluorescence in-situ hybridisation, *G1/2/3* grade 1/2/3, *IDH1/2* isocitrate dehydrogenase ½, *lncRNA* long non-coding RNA, *MALDI-TOF* MS matrix-assisted laser desorption/ionization time-of-flight mass spectrometry, *miRNA* microRNA, *MPNST* malignant peripheral nerve sheath tumour, *NFKBIA* Nuclear Factor Kappa B Inhibitor Alpha, *NGS* next generation sequencing, *NTA* nanoparticle tracking analysis, *OCEBM* Oxford Centre of Evidence-based Medicine, *OS* overall survival, *PD-L1* programmed death-ligand 1, *PFS* progression-free survival, *RAD21* RAD21 Cohesin Complex Component, *RMS* rhabdomyosarcoma, *RNA* ribonucleic acid, *RT-qPCR* real time quantitative polymerase chain reaction, *SERS* surface-enhanced Raman spectroscopy, *shWGS* shallow whole genome sequencing, *STS* soft tissue sarcoma, *STUMP* smooth muscle tumour of uncertain malignant potential, *TERT* telomerase reverse transcriptase, *TP53* tumour protein 53, *ULP-WGS* ultra-low passage whole genome sequencing, *WDLPS* well-differentiated liposarcoma

^a^Patients with ctDNA analysis available

^b^Results on cell lines not reported

Xie et al*.* [50] analysed circulating miR-26a-5p in 243 osteosarcoma patients. High miR-26a-5p levels as quantified with RT-qPCR allowed for differentiation between osteosarcoma and healthy controls (*N* = 96) [50]. Patients with “high” miR-26a-5p expression levels rather presented with metastatic or advanced disease and had worse overall survival (irrespective of clinical stage or distant metastases) than those with “low” ones [50].

Fujiwara et al*.* [51], analysed blood samples of 10 osteosarcoma patients, 10 non-osteosarcoma patients, and 10 healthy controls for miR-25-3p. Based on RT-qPCR, miR-25-3p was expressed at significantly higher levels in osteosarcoma than in non-osteosarcoma patients and healthy controls [51]. Osteosarcoma patients with “low” expression levels had a significantly longer metastasis-free survival [51].

Similarly, Wang et al*.* [52] quantified miR-491 levels with RT-qPCR in blood samples of 102 osteosarcoma patients. In comparison to 20 healthy controls, circulating miR-491 levels were significantly lower in osteosarcoma. Grouped into “high” and “low” circulating miR-491 levels, “low” levels predicted poor chemotherapeutic response, metastasis development, and worse overall survival [52].

Yang et al*.* [53], compared plasma samples of 42 osteosarcoma patients with those of 42 healthy controls for the presence of long non-coding RNA (lncRNA) MT1JP. Based on RT-qPCR results, plasma MT1JP levels were significantly lower in osteosarcoma patients than in controls [53].

Cahn et al*.* used the Foundation One^®^liquid CDx NGS panel to analyse various molecular alterations in paediatric and adolescent malignancies [24]. Positive cfDNA was detected in 4/7 osteosarcoma plasma samples analysed [24].

George et al*.* [17], used a ctDNA-specific NGS panel towards mutations identified on primary tumour tissue in one patient with osteosarcoma. Despite a delay of 1.4 years between primary tumour resection and acquisition of the plasma sample, one *TP53* mutation discovered in the original FFPE material was likewise detectable in plasma [17].

Zhang et al*.* [54] used fluorescence in-situ hybridisation (FISH) in peripheral blood samples of 23 osteosarcoma patients (13 recurrent/metastatic) analysing towards presence of CTCs. They found a higher number of CTCs in recurrence or metastasis in comparison to localised disease [54]. Moreover, ≥ 2 CTCs per 7.5 ml peripheral blood suggested shorter progression-free survival [54].

Bao et al. [55] analysed EVs with RNA sequencing in matched primary and metastatic blood plasma samples of two osteosarcoma patients, as well as blood plasma samples of a patient with primary osteosarcoma, and one with metastatic osteosarcoma of similar patient and primary tumour characteristics. A significant increase in tumour mutational burden of analysed EVs was discovered comparing non-metastatic to metastatic material [55].

Han et al*.* [56], studied plasma samples of 20 osteosarcoma patients and 20 healthy controls for CD63, vimentin and EpCAM levels. Compared with healthy controls, osteosarcoma patients displayed significantly higher levels of the three markers on plasma exosomes [56]. Using the same three markers within a diagnostic panel, osteosarcoma cases were identified with a sensitivity, specificity, and accuracy of 100%, 90%, and 95%, respectively [56].

In another study, the same group applied matrix-assisted laser desorption/ionization time-of-flight mass spectrometry (MALDI-TOF MS) to differentiate between osteosarcoma patients (*N* = 40) and healthy controls (*N* = 12), as well as metastatic (*N* = 20) and localised osteosarcoma patients (*N* = 20) based on plasma exosomes [57]. MALDI-TOF MS analysis allowed for differentiation between healthy controls and osteosarcoma patients, as well as osteosarcoma patients with localised and metastatic disease [57]. Additionally, a third study by the same group revealed that combination of MALDI-TOF MS and surface-enhanced Raman spectroscopy (SERS) for exosome profiling improves accuracy to discriminate osteosarcoma patients (*N* = 15) from healthy controls (*N* = 15) [58].

Wang et al*.* [59] measured circulating exosomal programmed death-ligand 1 (PD-L1) concentration in blood samples of 67 osteosarcoma patients using immunogold labelling and enzyme-linked immunosorbent assay (ELISA). Osteosarcoma patients with “high” exosomal PD-L1 concentration had a significantly poorer disease-free and overall survival [59].

#### Chondrosarcoma

We identified only two studies that exclusively investigated the potential of LB in chondrosarcoma. Both highlight the applicability of LB as diagnostic and prognostic markers in chondrosarcoma (Table 2).

Gutteridge et al*.* [60] analysed ctDNA levels in 29 chondrosarcoma patients based on a ddPCR assay for 5 different *isocitrate dehydrogenase 1* (*IDH1*) mutations. ctDNA was detected in 0% (0/6), 47.1% (8/17), 100% (2/2) and 100% (4/4) of G1, G2, G3 and dedifferentiated chondrosarcomas, respectively [60]. In patients with pre- and postoperative samples available, significant reduction in ctDNA levels was observed in 77.8% (7/9) following surgery, mirroring reduced tumour burden [60].

In another study, the same group [61] used the ddPCR assay to analyse ctDNA in pre- and postoperative blood samples of 83 chondrosarcoma patients with known *IDH1/2* or *GNAS* hotspot mutations. They detected ctDNA in 37% (31/83) of preoperative and 39% (12/31) of postoperative blood samples [61]. Again, presence of ctDNA correlated with tumour grade [61]. Presence of pre- or postoperative ctDNA was independently associated with poor overall survival [61].

#### Liposarcoma

In myxoid liposarcomas, a recurrent translocation leads to a fusion of the *CHOP* gene with *FUS* or *EWS*, potentially serving as a LB marker [62]. Well- and dedifferentiated liposarcoma (WDLPS/DDLPS) frequently harbour *MDM2* ampflications, likewise useful upon LB [63]. We identified 5 studies analysing the applicability of LB in different liposarcoma subtypes (Table 2).

Kohama et al*.* [64] discovered 3 miRNAs as diagnostic markers in the blood of 17 patients with DDLPS, amongst other sarcomas.

Fricke et al. [65] found two miRNAs with RT-qPCR significantly upregulated in DDLPS (*N* = 6) in comparison to healthy controls (*N* = 4). Yet, upon validation, only one miRNA remained significantly upregulated in DDLPS in comparison to healthy controls or lipoma patients (*N* = 5) [65].

Przybyl et al*.* [66], analysed cfDNA with shWGS towards presence of *MDM2* amplification in 3 patients with DDLPS and one patient with WDLPS. Whilst *MDM2* amplifications were found in cfDNA of 2/3 DDLPS patients, no amplification was present in the patient with WDLPS, or 11 patients with other soft tissue tumour types [66].

Braig et al*.* [67], found that analysis of *MDM2* amplifications in cfDNA was not sensitive enough to detect tumours in patients with WDLPS or DDLPS (*N* = 5). Notably, breakpoint t(12;16) and telomerase reverse transcriptase (TERT) C228T ctDNA levels as assessed with ddPCR correlated with clinical course and tumour burden in 4 patients with myxoid liposarcoma [67].

Jung et al*.* [68] analysed plasma samples of 20 patients with DDLPS undergoing treatment with an MDM2-inhibitor within the SAR405838 study towards presence of *TP53* mutations in cfDNA. In accordance with primary tumour material, none of the baseline blood samples revealed any *TP53* mutations, whilst upon MDM2-inhibitor treatment, multiple *TP53* were detected in cfDNA, suggesting emergence of *TP53* mutant clones resistant to MDM2 inhibition [68].

#### Leiomyosarcoma

Leiomyosarcomas have a complex karyotype, with several chromosomal losses and gains [69]. Thus, multiple CNAs can be used to identify ctDNA in leiomyosarcomas, as shown in two studies identified in this systematic literature review [70, 71]. A third study assessed the diagnostic potential of miRNAs to distinguish between benign uterine neoplasms and uterine sarcomas [72] (Table 2).

Yokoi et al*.* [72], analysed blood samples of 29 patients with uterine tumours towards circulating miRNAs using a miRNA microarray. Expression profiles of two miRNAs allowed for discrimination between uterine sarcomas and uterine leiomyomas [72].

Hemming et al*.* [70] analysed ctDNA in 30 patients with predominantly uterine leiomyosarcoma using ULP-WGS and ichorCNA. Of 16 patients with total tumour burden > 5 cm and progressive disease, 11 had detectable ctDNA (68.8%) [70]. No patient with stable disease or low tumour burden had measurable ctDNA levels [70].

Accordingly, Madanat-Harjuoja et al*.* [71] discovered that detectable ctDNA levels in 98 leiomyosarcoma patients enrolled in the SARC021 trial were associated with worse response, and poorer overall survival. They found ctDNA in 48/98 patients with pre-treatment samples available (49.0%), and in 17/69 (24.6%) patients with samples obtained after two chemotherapy cycles [71]. Presence of ctDNA in the latter group was associated with poorer overall survival [71].

#### Malignant peripheral nerve sheath tumour

Patients with neurofibromatosis type 1, develop multiple neurofibromas, plexiform neurofibromas, and eventually malignant peripheral nerve sheath tumours (MPNST) [73]. Differentiation between the latter two entities is essential, as malignant transformation from plexiform neurofibromas to MPNST has significant therapeutic and prognostic implications [74]. We found two LB studies dealing with this question Table 2.

Mattox et al. [74] did an aneuploidy analysis of ctDNA in blood stream of patients with neurofibromas (*N* = 7), plexiform neurofibromas (*N* = 9), and MPNS (*N* = 12). With a sensitivitiy of 30%, they identified MPNSTs out of the benign/intermediate tumours [74]. When adding sub-chromosomal CNA-analysis, sensitivity increased to 50% [74].

Szymanski et al*.* [75] aimed at identifying plexiform neurofibromas from their malignant counterparts, MPNST. ULP-WGS was used to detect cfDNA in blood of patients with plexiform neurofibromas (*N* = 23), MPNST (*N* = 14), and heathy controls (*N* = 16). MPNST patients had shorter cfDNA fragments and were enriched for tumour-derived cfDNA in comparison to patients with plexiform neurofibromas and healthy controls [75].

#### Myxofibrosarcoma

The complex karyotype of myxofibrosarcomas impedes detection of recurrent mutations via a LB approach. Unsurprisingly, we identified only one study investigating miRNAs as LB markers exclusively in myxofibrosarcomas [76] (Table 2).

Morita et al*.* identified miRN-1260b in the blood stream of myxofibrosarcoma patients as a diagnostic marker to distinguish them from healthy controls. Compared to the latter, miR-1260b levels were significantly higher in myxofibrosarcomas and correlated with a typical radiological sign (“tail sign”) found upon magnetic resonance imaging [76].

### Mixed sarcoma cohorts

Some authors collectively investigated LB approaches in different sarcoma subtypes, eventually allowing generalisability of results over several entities (Table 3).

**Table 3:** Studies reporting on use of blood-based liquid biopsy in mixed sarcoma cohorts

Study (alphabetical order)	Disease (patient number)	Material	Analysis	Relevance	
Asano N 2019 Nat Commun [85]	**Sarcoma (*N* = 414)** Intermediate soft tissue/bone tumour (*N* = 144) Benign soft tissue/bone tumour/healthy control (*N* = 339)	miRNA	RT-qPCR	miRNA profile of sarcoma patients different from the one of patients with benign bone/soft tissue tumours or healthy controls; molecular detector containing 7 miRNAs (Index VI) could distinguish sarcoma patients from benign tumours and healthy controls with 90% sensitivity and 95% specificity	III
Braig D 2022 Int J Mol Sci [83]	**STS (*N* = 3)**	ctDNA	Targeted NGS	ctDNA levels correlate with tumour burden; no ctDNA detectable upon complete disease remission; patients with later recurrence have ctDNA detectable at low thresholds, indicative of minimal residual disease	IV
Cahn F 2022 Cancers [24]	**STS (*N* = 9)** Osteosarcoma (*N* = 7) ES (*N* = 5) Other paediatric cancers (*N* = 24)	cfDNA	Foundation One®liquid CDx NGS panel (F1LCDx; 324 genes)	Specific mutations detectable in cfDNA from whole blood of ES (80%), osteosarcoma (57%) and STS (33%) patients	III
De Vos L 2020 J Mol Diagn [80]	**Sarcoma (*N* = 9)** Other cancer (*N* = 106)	cfDNA	RT-qPCR	Positive cumulative cfDNA methylation score (CMS; calculated based on *SHOX2* and *SEPT9* methylation status) found in 22.2% (2/9) of sarcoma patients; over all malignancies included, positive CMS present in 60.9% (70/115)	II
Demoret B 2019 Cancers [82]	**Leiomyosarcoma (*N* = 6)** **UPS (*N* = 6)** **DDLPS (*N* = 6)** **GIST (*N* = 6)**	ctDNA	ctDNA CGP panel	ctDNA detectable in 18/24 patients; subject level concordance rates for all overlapping genes were: 4/6 leiomyosarcoma patients, 2/6 UPS patients, 1/6 DDLPS patients, and 0/6 GIST patients; poor concordance for copy-number alterations	III
Eastley N 2020 Int J Mol Sci [81]	**STS (*N* = 29)**	cfDNA, ctDNA	Amplicon panel, IonTorrent AmpliSeq panel (Sarcoma V2) for SNVs, ddPCR	With Amplicon panel for *TP53, RB1* and *ARTX* mutations, ctDNA was found in 1/40 serial samples analysed (from 9 patients); ddPCR and IonTorrent AmpliSeq panel (Sarcoma V2) detected ctDNA in 17%; no correlation between intraoperative cfDNA levels and tumour size, histological subtype, development of later recurrence; no significant drop in cfDNA levels from pre- to postoperative	III
Heinhuis KM 2020 Cancers [77]	**Active sarcoma (*N* = 57)** Former sarcoma (*N* = 38) Healthy control (*N* = 65)	TEP-RNA	RNA sequencing	TEP-RNA profile significantly different in active sarcoma patients as compared with former sarcoma patients and healthy controls	III
McConnell L 2020 Cancers [79]	**Metastatic sarcoma (*N* = 12)**	cfDNA	NGS panel	Structural variants (5 fusion genes: *EWSR1::ATF1*, *MEAF6::PHF1, PAX3::FOXO1, EWSR1::FLI1, SS18::SSX2; 1 amplification: MDM2*) detected in 11/12 (91.6%) and 6/12 (50.0%) of tissue and plasma samples, respectively	IV
Namløs HM 2017 BMC Cancer [84]	**Spindle cell sarcoma (*N* = 1)**	ctDNA	Targeted resequencing (NCGC 900 cancer gene panel)	High ctDNA levels measured preoperatively; drop in ctDNA levels 3 days post-surgery; 6 weeks postoperatively, dramatic increase in ctDNA levels, corresponding to clinically evident multiple metastases	V
Pastuszak K 2021 Mol Oncol [78]	**Active sarcoma (*N* = 62)** Former sarcoma (*N* = 37) Healthy control (*N* = 75)	TEP-RNA	imPlatelet (RNA-based diagnostics), deep neural network	imPlatelet allows for discrimination between sarcoma patients and healthy controls with a balanced accuracy of 87% (compared to 100% for non-small cell lung cancer, and 91% for ovarian cancer)	III

The disease group of interest is highlighted in bold

*AFT1* activating transcription factor 1, *ATRX* alpha thalassemia/mental retardation syndrome x-linked, *CGP* comprehensive genomic profiling, *CGP* comprehensive genomic profiling, *ctDNA* circulating tumour deoxyribonucleic acid, *DDLPS* dedifferentiated liposarcoma, *ddPCR* digital droplet polymerase chain reaction, *GIST* gastrointestinal stromal tumour, *MEAF6* MYST/Esa1 associated factor 6, *OCEBM* Oxford Centre of Evidence-based Medicine, *PHF1* PHD finger protein 1, *RB1* retinoblastoma 1, *RNA* ribonucleic acid, *SEPT9* septin 9, *SHOX2* Short stature homeobox 2, *SNV* single nucleotide variant, *STS* soft tissue sarcoma, *TEP* tumour-educated platelet, *UPS* undifferentiated pleomorphic sarcoma

Based on the knowledge that blood platelets contain mRNAs that can undergo alternative splicing as a response to external stimuli, Heinhuis et al*.* [77] investigated the RNA profile of tumour-educated platelets (TEPs) in 57 sarcoma patients. Compared to healthy controls (*N* = 65) and former sarcoma patients (*N* = 38), TEP-RNA profiles were significantly different in active sarcoma [77].

Similarly, Pastuszak et al*.* [78], investigated TEP-RNA as a potential LB marker. A deep-learning image-based classifier (imPlatelet), converting RNA-sequenced platelet data from blood samples into images was used to differentiate between active sarcoma patients (*N* = 62) and former sarcoma patients (*N* = 37) or healthy controls (*N* = 75). Active sarcoma patients could be distinguished from the latter two with a balanced accuracy of 87% [78].

McConnell et al*.* [79] analysed cfDNA in peripheral blood of 12 sarcoma patients using NGS, detecting structural variants in 50.0% (6/12) of plasma samples, including 5 fusion genes (Table 3) and one *MDM2* amplification [79].

Cahn et al*.* [24] used the Foundation One^®^liquid CDx NGS panel to detect mutant cfDNA in peripheral blood of different sarcoma patients, including 9 with STS in 3 (33%) of which mutant cfDNA could be identified [24].

de Vos et al*.* [80], calculated a cumulative cfDNA methylation score (CMS) based on *Short stature homeobox 2* (*SHOX2*) and *septin 9* (*SEPT9*) methylation status in blood samples of 115 cancer patients, including 9 with sarcoma. Positive CMS (defined as CMS > 0.16%) was present in 60.9% of all entities, with a rate of 22.2% in sarcomas (2/9) [80].

Eastley et al. [81] analysed perioperative and follow-up blood samples of 29 non-metastasised STS patients towards cfDNA and ctDNA. Intraoperative cfDNA levels did not correlate with tumour size, histological subtype, or later development of local recurrence [81]. The amplicon-based approach targeting three common STS mutations detected mutant ctDNA in 1/40 serial samples analysed [81]. By using ddPCR and an IonTorrent AmpliSeq panel (Sarcoma V2), ctDNA was found in 17% of patients [81].

Demoret et al. [82], found ctDNA in 18/24 STS samples using comprehensive genomic profiling (CGP). Subject level concordance rates in all overlapping genes between tumour and LB samples were 66.7% (4/6), 33.3% (2/6), 16.7% (1/6) and 0% (0/6) for leiomyosarcoma, UPS, DDLPS, and GIST, respectively [82].

Braig et al*.* [83] used patient-specific mini-panels for targeted NGS, analysing serial ctDNA levels of 3 STS patients. No ctDNA was found upon permanent clinical remission, whilst patients with later recurrence presented with detectable ctDNA, indicative of minimal residual disease [83].

Namløs et al. [84], reported peri- and postoperative ctDNA dynamics in blood samples using targeted resequencing (NCGC 900 cancer gene panel) in a patient with primary localised spindle cell sarcoma. A postoperative drop in ctDNA levels was observed, followed by a dramatic increase after 6 weeks, corresponding to multiple metastases detected upon imaging [84].

Asano et al*.* [85] compared miRNA profiles of primary bone/STS patients (*N* = 414) with those of intermediate or benign bone/soft tissue tumour patients (*N* = 144) and healthy controls (*N* = 339). Sarcoma patients had significantly different miRNA profiles and a molecular detector (*Index VI*), based on serum levels of 7 miRNAs, differentiated between sarcoma patients and intermediate/benign tumour patients or healthy controls with a sensitivity and specificity of 90% and 95%, respectively [85].

## Discussion

This systematic literature review indicates that LB holds potential as a promising diagnostic and prognostic tool in different sarcoma entities. Although no clear line can be drawn between preclinical application of LB, including investigation of clonal evolution and metastatic behaviour, and its clinical use as a prognostic, predictive and diagnostic tool, this systematic literature review focused on the latter, wherefore experimental studies are only marginally discussed.

**Fig. 2:**
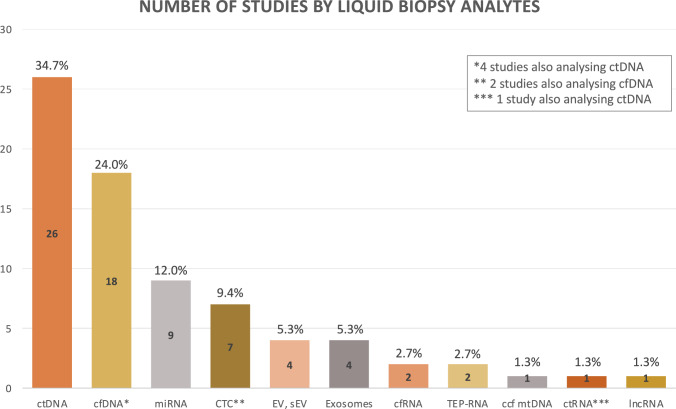
Number of studies by liquid biopsy analytes investigated in bone and soft tissue sarcomas

Especially the recurrent tumour-specific driver mutations as present in ES, GIST, and alveolar RMS enable the reliable detection and allow their longitudinal monitoring in the blood stream [13, 18]. The time-dependent behaviour of LB markers frequently corresponds with tumour burden [36, 42], treatment response [7, 20, 45] and relapse [6, 18, 20]. Overall, 42 of 68 studies investigated cfDNA and/or ctDNA as LB sources (and two additional studies used CTCs and cfDNA), reflecting the trend of using this analyte for LB in sarcoma (Fig. 2) [1].

However, also analysis of CTCs [29, 30, 54, 66], miRNAs [50–52, 64, 72, 76, 85] and TEP-RNA [77, 78] revealed partially promising results in diagnosis and prognosis assessment in different sarcomas. Notably, in sarcoma subtypes with tumour-specific mutations like ES and GIST, LB seems to be of special value given the high number of circulating analytes. Conversely, studies on LB in synovial sarcoma, another translocation-associated mesenchymal neoplasm, revealed a relatively low prevalence of tumour-specific fusion transcripts detectable with LB. Therefore, further in-depth research on tumour-tailored adjustment of methods and analytes will be required to identify the most suitable LB approach depending on histological subtype and research question. To date, the major limitation of LB in sarcoma constitutes its variable clinical utility depending on underlying histology. As another limitation, the technical and logistic requirements associated with most LB approaches hinder deployment of a well-functioning clinical workflow. Moreover, a standardisation of liquid biopsy approaches—ideally irrespective of the histological subtype—should be strived for to allow for comparability between studies. In line with this, the timing of liquid biopsy measurements, both prior, during and after therapy, should follow a consistent protocol, ideally aligning with (neo)adjuvant treatment regimens and clinical/radiological follow-up appointments.

## Conclusions

Overall, the use of LB in sarcomas is increasing, constituting a non-invasive but powerful tool for diagnosis, prognostication and disease monitoring, thus eventually enabling faster tumour diagnosis, improving individual patients’ outcomes, reducing the need for follow-up imaging modalities (e.g. MRI, CT), and initiating/adapting therapeutic regimens at earlier time points. However, one has to bear in mind the varying clinical performance of LB depending on sarcoma subtypes. While LB achieves promising results in translocation-associated sarcomas, its clinical utility in complex-karyotype sarcomas is yet to be established.

## Supplementary Information

Below is the link to the electronic supplementary material.

Supplementary file1 (DOCX 154 kb)

## Data Availability

The data collected for this systematic review is available upon reasonable request from the corresponding author.
