# Supplementary material for: Precision medicine in diagnosis, prognosis, and disease monitoring of bone and soft tissue sarcomas using liquid biopsy: a systematic review
Source: Arch Orthop Trauma Surg. 2025 Jan 11;145(1):121. doi: 10.1007/s00402-024-05711-w (PMC11724793; doi:10.1007/s00402-024-05711-w)
Supplement: Supplementary file 1 — Supplementary file1 (DOCX 154 kb) [file 402_2024_5711_MOESM1_ESM.docx]

**SUPPORTING INFORMATION**

**Title**: Precision Medicine in Diagnosis, Prognosis, and Disease Monitoring of bone and soft tissue sarcomas using Liquid Biopsy. A systematic review.

**Supplementary Table 1** PubMed search code.

**Supplementary Figure 1** PRISMA flow-chart showing study selection process.

**Supplementary Table 1** PubMed search code.

| **Search Term** | **Query** |
| --- | --- |
| Liquid biopsy AND sarcoma | ("liquid biopsy"[MeSH Terms] OR ("liquid"[All Fields] AND "biopsy"[All Fields]) OR "liquid biopsy"[All Fields]) AND ("sarcoma"[MeSH Terms] OR "sarcoma"[All Fields] OR "sarcomas"[All Fields] OR "sarcoma s"[All Fields])  **Translations**  liquid biopsy: "liquid biopsy"[MeSH Terms] OR ("liquid"[All Fields] AND "biopsy"[All Fields]) OR "liquid biopsy"[All Fields]  sarcoma: "sarcoma"[MeSH Terms] OR "sarcoma"[All Fields] OR "sarcomas"[All Fields] OR "sarcoma's"[All Fields] |
| Liquid biopsy AND soft tissue sarcoma | ("liquid biopsy"[MeSH Terms] OR ("liquid"[All Fields] AND "biopsy"[All Fields]) OR "liquid biopsy"[All Fields]) AND ("sarcoma"[MeSH Terms] OR "sarcoma"[All Fields] OR ("soft"[All Fields] AND "tissue"[All Fields] AND "sarcoma"[All Fields]) OR "soft tissue sarcoma"[All Fields])  **Translations**  liquid biopsy: "liquid biopsy"[MeSH Terms] OR ("liquid"[All Fields] AND "biopsy"[All Fields]) OR "liquid biopsy"[All Fields]  soft tissue sarcoma: "sarcoma"[MeSH Terms] OR "sarcoma"[All Fields] OR ("soft"[All Fields] AND "tissue"[All Fields] AND "sarcoma"[All Fields]) OR "soft tissue sarcoma"[All Fields] |
| liquid biopsy AND bone sarcoma | ("liquid biopsy"[MeSH Terms] OR ("liquid"[All Fields] AND "biopsy"[All Fields]) OR "liquid biopsy"[All Fields]) AND ("osteosarcoma"[MeSH Terms] OR "osteosarcoma"[All Fields] OR ("bone"[All Fields] AND "sarcoma"[All Fields]) OR "bone sarcoma"[All Fields])  **Translations**  liquid biopsy: "liquid biopsy"[MeSH Terms] OR ("liquid"[All Fields] AND "biopsy"[All Fields]) OR "liquid biopsy"[All Fields]  bone sarcoma: "osteosarcoma"[MeSH Terms] OR "osteosarcoma"[All Fields] OR ("bone"[All Fields] AND "sarcoma"[All Fields]) OR "bone sarcoma"[All Fields] |
| liquid biopsy AND osteosarcoma | ("liquid biopsy"[MeSH Terms] OR ("liquid"[All Fields] AND "biopsy"[All Fields]) OR "liquid biopsy"[All Fields]) AND ("osteosarcoma"[MeSH Terms] OR "osteosarcoma"[All Fields] OR "osteosarcomas"[All Fields])  **Translations**  liquid biopsy: "liquid biopsy"[MeSH Terms] OR ("liquid"[All Fields] AND "biopsy"[All Fields]) OR "liquid biopsy"[All Fields]  osteosarcoma: "osteosarcoma"[MeSH Terms] OR "osteosarcoma"[All Fields] OR "osteosarcomas"[All Fields] |
| liquid biopsy AND ewing sarcoma | ("liquid biopsy"[MeSH Terms] OR ("liquid"[All Fields] AND "biopsy"[All Fields]) OR "liquid biopsy"[All Fields]) AND ("sarcoma, ewing"[MeSH Terms] OR ("sarcoma"[All Fields] AND "ewing"[All Fields]) OR "ewing sarcoma"[All Fields] OR ("ewing"[All Fields] AND "sarcoma"[All Fields]))  **Translations**  liquid biopsy: "liquid biopsy"[MeSH Terms] OR ("liquid"[All Fields] AND "biopsy"[All Fields]) OR "liquid biopsy"[All Fields]  ewing sarcoma: "sarcoma, ewing"[MeSH Terms] OR ("sarcoma"[All Fields] AND "ewing"[All Fields]) OR "ewing sarcoma"[All Fields] OR ("ewing"[All Fields] AND "sarcoma"[All Fields]) |
| liquid biopsy AND chondrosarcoma | ("liquid biopsy"[MeSH Terms] OR ("liquid"[All Fields] AND "biopsy"[All Fields]) OR "liquid biopsy"[All Fields]) AND ("chondrosarcoma"[MeSH Terms] OR "chondrosarcoma"[All Fields] OR "chondrosarcomas"[All Fields])  **Translations**  liquid biopsy: "liquid biopsy"[MeSH Terms] OR ("liquid"[All Fields] AND "biopsy"[All Fields]) OR "liquid biopsy"[All Fields]  chondrosarcoma: "chondrosarcoma"[MeSH Terms] OR "chondrosarcoma"[All Fields] OR "chondrosarcomas"[All Fields] |
| cell-free DNA AND sarcoma AND liquid biopsy | ("cell free nucleic acids"[MeSH Terms] OR ("cell free"[All Fields] AND "nucleic"[All Fields] AND "acids"[All Fields]) OR "cell free nucleic acids"[All Fields] OR ("cell"[All Fields] AND "free"[All Fields] AND "dna"[All Fields]) OR "cell free dna"[All Fields]) AND ("sarcoma"[MeSH Terms] OR "sarcoma"[All Fields] OR "sarcomas"[All Fields] OR "sarcoma s"[All Fields]) AND ("liquid biopsy"[MeSH Terms] OR ("liquid"[All Fields] AND "biopsy"[All Fields]) OR "liquid biopsy"[All Fields])  **Translations**  cell-free DNA: "cell-free nucleic acids"[MeSH Terms] OR ("cell-free"[All Fields] AND "nucleic"[All Fields] AND "acids"[All Fields]) OR "cell-free nucleic acids"[All Fields] OR ("cell"[All Fields] AND "free"[All Fields] AND "dna"[All Fields]) OR "cell free dna"[All Fields]  sarcoma: "sarcoma"[MeSH Terms] OR "sarcoma"[All Fields] OR "sarcomas"[All Fields] OR "sarcoma's"[All Fields]  liquid biopsy: "liquid biopsy"[MeSH Terms] OR ("liquid"[All Fields] AND "biopsy"[All Fields]) OR "liquid biopsy"[All Fields] |
| cell free DNA AND sarcoma AND biopsy | ("cell free nucleic acids"[MeSH Terms] OR ("cell free"[All Fields] AND "nucleic"[All Fields] AND "acids"[All Fields]) OR "cell free nucleic acids"[All Fields] OR ("cell"[All Fields] AND "free"[All Fields] AND "dna"[All Fields]) OR "cell free dna"[All Fields]) AND ("sarcoma"[MeSH Terms] OR "sarcoma"[All Fields] OR "sarcomas"[All Fields] OR "sarcoma s"[All Fields]) AND ("biopsie"[All Fields] OR "biopsy"[MeSH Terms] OR "biopsy"[All Fields] OR "biopsied"[All Fields] OR "biopsies"[All Fields] OR "biopsy s"[All Fields] OR "biopsying"[All Fields] OR "biopsys"[All Fields] OR "pathology"[MeSH Subheading] OR "pathology"[All Fields])  **Translations**  cell free DNA: "cell-free nucleic acids"[MeSH Terms] OR ("cell-free"[All Fields] AND "nucleic"[All Fields] AND "acids"[All Fields]) OR "cell-free nucleic acids"[All Fields] OR ("cell"[All Fields] AND "free"[All Fields] AND "dna"[All Fields]) OR "cell free dna"[All Fields]  sarcoma: "sarcoma"[MeSH Terms] OR "sarcoma"[All Fields] OR "sarcomas"[All Fields] OR "sarcoma's"[All Fields]  biopsy: "biopsie"[All Fields] OR "biopsy"[MeSH Terms] OR "biopsy"[All Fields] OR "biopsied"[All Fields] OR "biopsies"[All Fields] OR "biopsy's"[All Fields] OR "biopsying"[All Fields] OR "biopsys"[All Fields] OR "pathology"[Subheading] OR "pathology"[All Fields] |

**Supplementary Figure 1** PRISMA flow-chart showing study selection process.
